# A survey of health literacy among household health ambassadors in Azarbaijan province, Iran

**DOI:** 10.34172/hpp.025.44368

**Published:** 2025-07-15

**Authors:** Parvin Reyhani, Leili Faraji, Hannaneh Reyhani, Kamyar Pirehbabi, Leila Zhianfar, Farzaneh Golboni

**Affiliations:** ^1^Department of Health Education and Promotion, Faculty of Health, Tabriz University of Medical Sciences, Tabriz, Iran; ^2^Department of Statistics and Epidemiology, Faculty of Health, Tabriz University of Medical Sciences, Tabriz, Iran; ^3^Health Center of Tabriz, Tabriz University of Medical Sciences, Tabriz, Iran; ^4^Department of Nursing, Faculty of Nursing and Midwifery, Mazandaran University of Medical Sciences, Sari, Iran; ^5^Farzaneh Golboni, Assistant Professor at the Ministry of Health and Medical Education (MOHME), Tehran, Iran

**Keywords:** Health education, Health literacy, Trained health volunteers

## Abstract

**Background:**

Health literacy (HL) plays a crucial role in how people make decisions in their daily living activities. Considering the critical role of HL among household health ambassadors (HAs) in promoting their health and that of their households, this study aimed to examine the HL of household HAs in Iran.

**Methods:**

In this HL survey, a number of 2183 household HAs from 18 cities of East Azarbaijan province was recruited to participate in the study. The data were collected applying the Health Literacy for Iranian Adults (HELIA) Questionnaire via an online link, which were then analyzed applying multivariate linear regression with enter method by using SPSS version 19.

**Results:**

The results revealed an average HL score of 14.96 (SD 3.21). Among the participants, approximately 8% [95% confidence interval (CI): 5.13-11.62] (n=174) exhibited poor HL, while 41.2% [95% CI: 37.45-51.32] (n=899) demonstrated moderate and 50.8% [95% CI: 42.87-59.23] (n=1109) achieved good levels of HL.

**Conclusion:**

Our findings emphasized the urgent need to assess and enhance the HL of household HAs in a developing country, like Iran. Notably, about half of participants exhibited poor or moderate levels of HL. This trend underscores the vital role that HAs play in promoting HL and facilitating individual self-care within their communities. To empower these ambassadors, it is crucial to implement innovative and targeted strategies that effectively boost their HL.

## Introduction

 Health literacy (HL) is a critical component of public health,^1,2^ and a priority in public health policies.^3^ According to the World Health Organization, HL refers to the cognitive and social skills that determine individuals’ motivation and ability to obtain, understand, and use health information for health maintenance and improvement of their health.^4^ As a global issue,^5^ HL was emphasized at the 5th World Health Promotion Conference in Mexico as an individual characteristic and a key determinant of health and well-being at the community level.^6-8^ HL encompasses a set of skills to read, write, count, communicate, and use electronic technology, as well as to make appropriate health decisions.^9,10^ additionally, it is one of the social components of health and can play an important role in empowering individuals to overcome health obstacles,^11^ change their environment, and influence health. The health system should provide the necessary structure and facilities to promote HL at the community level.^12^

 Improving HL leads to greater independence and empowerment, better quality of life, justice and sustainability of changes in public health within societies,^13,14^ Conversely, low HL is associated with significant inequality and high costs within the healthcare system, making it a concern for policymakers.^3^ Data indicate that more than one-third of U.S. adults have limited HL, which adversely affects patient safety, access to care, and overall quality of healthcare.^10,15^ Most countries in the world have adopted national policies and programs to increase HL. In recent decades, the connection between insufficient HL and poor health status, poor preventive behaviors, and high costs of health care services, and on the other hand, the impact of HL on social determinants of health and its overall effect on health status has been recognized.^16-20^

 People need health information to maintain their own health and that of their families, which can be enhanced through the participation of patients and their families in health care systems. The Health Literacy Research and Practice Unit brings together diverse fields, including education, health care, social sciences, and cultural sciences, along with organizations dedicated to promoting HL.^21^ Members of the community face challenges when searching for health-related information due to the complexities of the health care system and the overwhelming amount of available information.^22^

 The concept of health ambassadors (HAs) has gained traction as a supportive strategy in enhancing public HL. Research highlights the effectiveness of HAs in various settings, from rural communities to urban environments, demonstrating their role in increasing access to health information and promoting healthier lifestyles. For instance, studies conducted in countries like the United States^23^ and Australia^24^ indicate that HAs not only facilitate understanding of health information but also empower individuals to make informed health decisions, leading to improved health outcomes. Despite this growing body of evidence, there remains a gap in understanding the specific contributions of HAs in different cultural contexts, particularly in developing countries.

 In Iran, the deployment of HAs has been a significant step towards addressing public health challenges and improving HL. Previous studies have emphasized their potential impact on community health,^25^ but there is limited empirical research examining the HL levels among these ambassadors. Despite the recognized important role of HAs in improving community HL globally, there is a notable paucity of empirical data on their HL levels, particularly within developing countries and specific regions such as Iran’s East Azarbaijan province. While studies have demonstrated the effectiveness of HAs in facilitating health information and promoting healthier behaviors, understanding the literacy competence of these individuals remains limited. This gap is critical because the success of HAs in health promotion heavily depends on their ability to comprehend and communicate health information effectively. Addressing this knowledge void is essential for designing targeted training programs and enhancing the overall impact of HAs, ultimately contributing to the development of more effective community-based HL interventions.

 Given the crucial role of HAs as facilitators of HL, this study aims to assess their HL levels and identify areas for improvement. By focusing on this under-researched area, the study seeks to provide actionable recommendations for enhancing the capacities of HAs, ultimately aiming to promote better health outcomes within the region.

## Materials and Methods

###  Participants

 The current study was a survey conducted among household HAs in 18 cities in East Azarbaijan province, Iran. A multistage sampling approach was used to select participants, which included stratified and cluster sampling methods. First, the cities were stratified based on relevant demographic or administrative characteristics. Within each city, clusters were defined as specific districts or neighborhoods, from which households were systematically selected. In the final stage, simple random sampling was applied to select individual household HAs who met the inclusion criteria. The sample size was calculated to be 2,183 using the Cochran formula, taking into account the desired confidence level and margin of error. Inclusion criteria included being registered as a household HA within the provincial health system and providing informed consent to participate in the study.

###  Instruments and procedure

 The data collection tools included demographic information and the electronic health literacy questionnaire for Iranian adults (HELIA). The demographic questionnaire included age, gender, educational level, place of residence, and employment status.

 The HELIA has been validated and deemed reliable in Iran.^26^ Although test-retest was not reported in the initial study, Cronbach’s alpha for the domains of the scale was found to be ranged from 0.72 to 0.89. This questionnaire consisted of 33 items assessing household HAs across five dimensions of HL. These dimensions were access (six items), reading (four items), comprehension (seven items), evaluation (four items), and decision-making (12 items). Each item was rated on a 5-point Likert scale, with responses ranging from 1 (never) to 5 (always). To determine the HL score, the weight of each section was calculated based on the score obtained in that section, divided by the number of items. Then, we divided the total weighted scores of all subgroups by 5 and classified them into three categories (poor literacy: ≤ 10, moderate literacy 11-15, good literacy: > 15).^27^

 Data were collected using an online questionnaire developed in the Porsline system (https://porsline.ir) and administered by the HAs. To approach participants for the online data collection, health educationists in the health system of the province were trained to effectively engage HAs and explain the purpose of the study, emphasizing the importance of their participation in this study. The health educationists utilized a variety of communication methods, including phone calls, social media platforms, and in-person interactions, to inform potential respondents about the online questionnaire hosted on the Porsline system. Participants were provided with clear instructions on how to access and complete the questionnaire, along with assurances of confidentiality to encourage honest responses. To maximize participation, the health educationists followed up with reminders and addressed any questions or concerns from participants, ensuring a thorough and respectful collection of data. All official approvals and agreements were obtained from the Local authorities in the district headquarters of Health and Medical Education (MOHME) before the study commenced.

###  Statistics

 The collected data were analyzed using descriptive statistics, frequency distribution tables, statistical tests of Pearson’s correlation coefficient, and multivariate linear regression using IBM SPSS Statistics version 19 software (IBM Corp., Armonk, NY, USA). For normality, the distribution of continuous variables was checked using the Shapiro-Wilk test and visual inspections of histograms and Q-Q plots. Scatterplots were examined to confirm linear relationships between continuous predictors and outcomes. Cases with extreme values were identified through boxplots and standardized residuals. Missing data were addressed through multiple imputation, ensuring that the assumptions associated with this technique were satisfied. The analysis accounted for the sampling strategy’s design effect, applying weighting or complex survey procedures, as advised by the statistician to ensure accurate inference.

 Potential confounders, including age, gender, and socioeconomic status, were included as covariates in the multivariate regression models to control their influence on the primary relationships of interest. The multivariate linear regression was performed using an enter method, with stepwise procedures as appropriate, based on the consultations with the statistician. The models were checked for multicollinearity, and interaction effects were tested where relevant. The significance level for all tests was set at 0.05.

## Results

 The results showed that the mean age of the household HAs (n = 2183) was 34.51 (SD 9.35) years. A majority of HAs were male (87.4%), diploma (33.3%), housewives (59.7%), and urban residents (51.1%). Overall, most participants demonstrated good HL, accounting for 50.8% [95% CI: 42.87-59.23] of the total population, while 41.2% [95% CI: 37.45-51.32] exhibited moderate HL, and 8.0% [95% Confidence Interval (CI): 5.13-11.62] were classified as having poor HL. When analyzed by gender, most female ambassadors (55.8%) [95% CI: 48.63-61.42] demonstrated good HL compared to male ambassadors, with 41.5% [95% CI: 37.41-51.12] exhibiting moderate literacy levels. Based on the findings, most of the HAs (n = 1109) had adequate HL (50.8%) [95% CI: 42.87-59.23] (Table 1).

**Table 1:** Frequency (percentage) and Health literacy levels of demographic variables of household health ambassadors in East Azarbaijan province

**Demographic characteristics**	**N (percent)**	**Poor HL** **n (%)**	**Moderate HL** **n (%)**	**Good HL** **n (%)**	***P*** ** value**
Total Population	2183 (100.0)	174 (8.0)	899 (41.2)	1109 (50.8)	< 0.001
Age mean (SD)	34.51 ± 9.35	34.63 ± 9.02	34.81 ± 9.45	34.25 ± 9.31	
Gender					< 0.001
Male	1907 (87.4)	160 (8.4)	792 (41.5)	955 (50.1)	
Female	274 (12.6)	107 (39.1)	153 (55.8)	208 (75.1)	
Education					< 0.01
Under diploma	614 (28.1)	101 (16.4)	290 (47.2)	223 (36.3)	
Diploma	727 (33.3)	53 (7.3)	322 (44.3)	352 (48.4)	
Post-diploma	154 (7.1)	5 (3.2)	54 (35.1)	95 (61.7)	
Bachelor's degree	687 (31.5)	15 (2.2)	233 (25.9)	687 (31.5)	
Place of residency					< 0.01
Urban	1114 (51.1)	55 (4.9)	424 (38.1)	635 (57.0)	
Rural	1068 (48.9)	119 (11.1)	475 (44.5)	474 (44.4)	
Employment					< 0.001
Government officer	425 (19.5)	9 (2.1)	125 (29.4)	291 (68.5)	
Teacher	92 (4.2)	2 (2.2)	29 (31.5)	61 (66.3)	
Self-employed	28 (1.3)	0 (0.0)	15 (53.6)	13 (46.4)	
Shopkeeper	34 (1.6)	1 (2.9)	14 (41.2)	19 (55.9)	
Worker	33 (1.5)	2 (6.1)	14 (42.4)	17 (51.5)	
Farmer	24 (1.1)	6 (25.0)	8 (33.3)	10 (41.7)	
Rancher	7 (0.3)	1 (14.3)	4 (57.1)	2 (28.6)	
Housewife	1303 (59.7)	143 (11.0)	592 (45.4)	568 (43.6)	
Others	237 (10.9)	10 (4.2)	98 (41.5)	128 (54.2)	

HL: health literacy; SD: standard deviation.

 According to the results presented in Table 2, participants had good HL in Understanding and Decision-making, but moderate HL in reading. The overall mean HL score was highest among females (15.49 ± 3.79) compared to males (14.82 ± 3.74) (*P* < 0.001). In terms of Education level, those holding a bachelor’s degree demonstrated the highest mean scores across all domains (16.45 for reading, 16.87 for access, etc), while individuals with education below a diploma had the lowest scores (12.24 for reading). Also, urban HAs achieved a mean score of 15.05 ± 4.11 compared to their rural counterparts, who had a lower mean of 13.43 ± 4.75, highlighting a potential urban-rural divide. Employment status further impacted HL levels, with employees reporting the highest mean scores (16.02 ± 3.67), while those in farming exhibited the lowest (11.56 ± 5.20) (*P* < 0.001). Furthermore, simple linear regression was used to assess differences in the HL scores by demographic characteristics. In all educational levels, as education increased, the HL score showed a significant increase compared to individuals with lower education (*P* < 0.001). Additionally, the HL scores for all employment categories were lower than those of employees (Table 2).

**Table 2:** Total score and mean of Health literacy in different domains of household health ambassadors in East Azarbaijan province

**Demographic**	**Mean Health literacy**					**Health literacy score**	**Unadjusted**		**Adjusted**	
**Characteristics**	**Reading,** **Mean±SD**	**Access,** **Mean±SD**	**Understanding,** **Mean±SD**	**Appraisal, Mean±SD**	**Decision-making, Mean±SD**		**β**	***P*** ** value**	**β**	***P*** ** value**
Age						14.96	-0.085	0.004	-0.055	0.009
Gender										
Male	14.21 ± 4.53	14.65 ± 3.72	15.71 ± 3.46	14.82 ± 3.74	15.18 ± 3.42	15.90	-	-	-	-
Female	14.61 ± 4.35	15.09 ± 3.86	16.07 ± 3.17	15.49 ± 3.79	15.85 ± 3.41	16.40	-0.015	0.123	-0.021	0.383
Education										
Under diploma	12.24 ± 4.90	13.30 ± 3.77	14.46 ± 3.70	13.62 ± 3.86	14.57 ± 3.59	14.60	-	-	-	-
Diploma	14.15 ± 4.28	14.64 ± 3.65	15.63 ± 3.35	14.84 ± 3.59	15.28 ± 3.37	15.90	0.197	< 0.001	0.159	< 0.001
Post-diploma	15.42 ± 3.88	15.51 ± 3.47	16.45 ± 2.96	15.83 ± 3.59	15.95 ± 3.21	16.83	0.163	< 0.001	0.124	< 0.001
Bachelor's degree	15.92 ± 3.70	15.85 ± 3.43	16.87 ± 2.89	15.92 ± 3.55	15.72 ± 3.27	17.05	0.289	< 0.001	0.227	< 0.001
Place of residency										
Urban	15.05 ± 4.11	15.15 ± 3.61	16.31 ± 3.16	15.37 ± 3.72	15.55 ± 3.22	16.48	-	-	-	-
Rural	13.43 ± 4.75	14.24 ± 3.82	15.17 ± 3.59	14.41 ± 3.75	14.97 ± 3.60	15.43	-0.090	0.002	-0.060	0.007
Employment										
Ggovernment officer	16.02 ± 3.67	16.33 ± 3.30	17.18 ± 2.80	16.39 ± 3.50	16.56 ± 3.03	17.49	-	-	-	-
Teacher	15.98 ± 3.47	16.21 ± 3.37	16.82 ± 2.78	16.06 ± 3.27	16.30 ± 2.81	17.27	-0.037	0.234	-0.021	0.346
Self-employed	14.24 ± 4.06	15.15 ± 3.85	15.76 ± 3.21	15.80 ± 3.82	15.42 ± 3.13	16.20	-0.041	0.103	-0.027	0.199
Shopkeeper	14.96 ± 4.86	15.91 ± 4.02	15.88 ± 3.66	16.10 ± 3.39	15.82 ± 3.44	16.67	-0.039	0.125	-0.026	0.219
Worker	14.54 ± 4.25	14.61 ± 3.70	15.73 ± 3.06	14.54 ± 3.52	15.59 ± 3.47	15.94	-0.051	0.087	-0.035	0.106
Farmer	11.56 ± 5.20	12.36 ± 4.15	13.93 ± 3.74	13.70 ± 3.41	14.08 ± 3.72	14.13	-0.085	< 0.001	-0.077	< 0.001
Rancher	10.71 ± 4.01	12.62 ± 4.31	12.75 ± 3.63	13.03 ± 2.69	14.17 ± 3.24	13.66	-0.074	< 0.001	-0.062	< 0.001
Housewife	13.52 ± 4.62	14.06 ± 3.64	15.18 ± 3.54	14.23 ± 3.71	14.76 ± 3.42	15.33	-0.311	< 0.001	-0.239	< 0.001
Others*	14.77 ± 4.42	14.89 ± 3.93	16.15 ± 3.11	15.45 ± 3.78	15.32 ± 3.61	16.33	-0.142	< 0.001	-0.090	< 0.001

SD: Standard Deviation; * others mean the jobs other than those presented in the table.

Table 3 illustrates the distribution of HL levels across various domains among the participants. A substantial proportion of HAs exhibited good HL, with 50.8% demonstrating proficiency in reading, while 41.2% were classified as having moderate HL, and only 8.0% falling into the poor HL category. Similar patterns were observed across the domains of availability, understanding, appraisal, and decision-making, with good HL consistently representing around 51.1% and moderate literacy approximately 41.1%. The prevalence of poor HL remained low across all domains, ranging from 7.6% to 8.0%.

**Table 3:** Frequency (percentage) of Health literacy levels based in different domains of household health ambassadors in East Azarbaijan province

**Domains of HL **	**Poor HL, n (%)**	**Moderate HL, n (%)**	**Good HL, n (%)**	***P*** ** value**
Reading	174 (8.0)	899 (41.2)	1109 (50.8)	< 0.001
Access	171 (7.9)	892 (41.1)	1109 (51.1)	< 0.001
Understanding	165 (7.6)	896 (41.3)	1107 (51.1)	< 0.001
Appraisal	174 (8.0)	899 (41.2)	1109 (50.8)	< 0.001
Decision-making	174 (8.0)	899 (41.2)	1109 (50.8)	< 0.001

HL: health literacy.

## Discussion

 The present study aimed to investigate the HL status and explore the influential factors among household HAs in 16 cities of East Azarbaijan province, Iran. Evaluating the HL of household HAs is vital, given their pivotal role in improving HL and supporting self-care within the community. In this study, HL was categorized into three levels: poor ( ≤ 10), moderate (11-15), and good ( > 15). The findings revealed a mean HL score of 14.96 ± 3.21, indicating that more than half of the household HAs fell within the moderate and good HL categories. This finding aligns with studies conducted by Damman et al,^28^ Rajabalipour et al,^29^ Aghajanloo et al,^30^ Ghanbari et al,^31^ and Jovic-Vranes & Bjegovic-Mikanovic,^32^ who similarly reported strong HL levels among various populations. However, the results also diverge from those of Yari et al,^27^ and Seyedoshohadaee et al,^33^ who noted lower HL levels in their respective studies. The discrepancies in HL scores observed across these studies may be attributed to differences in assessment tools and the demographic characteristics of the study populations. These variations also highlight the need for standardized measures and a more comprehensive understanding of the factors influencing HL across different communities.

 Our findings suggest that demographic factors such as age, gender, education, and employment status significantly affect HL, emphasizing the need for targeted interventions to address disparities among these groups.^22,27,34,35^ The results indicated that men had higher HL levels than women, aligning with some studies.^1,27,30,36,37^ However, Paasche-Orlow et al. reported that women had higher HL,^35^ which contradicts our findings. This discrepancy is further compounded by the findings of Khoshravesh et al.,^38^ who indicated no difference between genders across various dimensions of HL. These variations highlight the complexity of HL as it relates to gender and suggest that further investigation is warranted to understand the underlying factors influencing these differing outcomes.

 Our results showed that education is an important predictor of HL. These findings are consistent with those reported in several studies.^22,32,39-42^ Individuals with higher levels of education tend to possess a greater capacity to effectively evaluate health information, adopt beneficial health behaviors, adapt their environments, and utilize self-care services, all of which contribute positively to their health status. Furthermore, previous research has established a positive relationship between HL and better health outcomes,^32,43^ reinforcing the idea that education plays a critical role in enhancing individuals’ ability to navigate health-related challenges. These insights suggest that enhancing educational opportunities for HAs could be a crucial strategy for increasing HL within communities. By prioritizing education for these individuals, we enable them to gain the essential knowledge and skills needed to disseminate accurate health information. Empowering HAs through education will equip them to make informed health decisions and effectively engage with their communities. This empowerment not only improves their own HL but also enhances their capacity to influence the health outcomes of those they support, leading to healthier communities overall.

 Furthermore, the mean score of HL among urban residents was higher than those of rural residents in all regions, which is consistent with the study of Yari et al.^27^ This finding suggests that urban HAs benefit from greater access to health facilities, the presence of robust social institutions, and enhanced communication networks with these organizations. These factors provide urban HAs with more opportunities to acquire essential health information and skills. As a result, they are better equipped to develop effective behavioral and decision-making strategies regarding health. This highlights the importance of supporting rural HAs by improving access to resources and information, ultimately fostering HL in all communities.

 Our analysis indicates that HL scores tend to decrease with increasing age; however, this relationship was not statistically significant (*P* value = 0.35). Interestingly, some studies have identified a reverse relationship, suggesting that older individuals may possess higher HL levels.^34,41^ The findings of the current study align with those of Carthery-Goulart et al ^44^ and Safeer and Keenan,^45^ who observed similar trends. In contrast, the results reported by von Wagner et al. suggested a different perspective, reinforcing the complexity of the relationship between age and HL.^46^ The observed trend of declining HL with age may be linked to factors such as decreased cognitive function and a diminished ability to effectively utilize health information. These insights underscore the need for targeted educational interventions and support systems tailored to older HAs, ensuring they have the necessary resources to enhance their HL and continue contributing effectively to their communities.

 Employees and teachers consistently scored higher in all dimensions of HL, particularly within the “Understanding” category, compared to individuals in other job categories. This trend may be attributed to the impact of social factors, such as video media-based training on health issues, which effectively enhances their understanding and evaluation skills.^47^ Conversely, salespeople and marketers excelled in the Appraisal dimension, highlighting their proficiency in evaluating health information. In contrast, workers scored lower across all dimensions of HL, suggesting that they may face barriers related to educational attainment and lack access to resources that could improve their HL. These findings emphasize the need for targeted interventions to enhance HL among workers, ensuring they gain the knowledge and skills necessary to make informed health decisions. By addressing these gaps, we can foster a more health-literate workforce that contributes positively to overall community well-being.

 Overall, participants demonstrated commendable HL across all dimensions, with scores reported as follows: reading at 50.8%, access at 51.1%, understanding at 51.1%, appraisal at 50.8%, and decision-making at 50.8%. These results are consistent with several studies in the field,^28-32^ yet they contrast with the findings of Zareipour et al. ^3^ and Seyedoshohadaee et al. ^33^ The variations observed in HL scores among different studies may be attributed to discrepancies in measurement tools or differences in the demographic characteristics of the participants. These insights highlight the importance of standardizing assessment methods and considering the diverse backgrounds of participants when interpreting HL results. By understanding these factors, we can better contextualize HL outcomes and develop targeted strategies to improve HL across varied populations.

 To elucidate the mechanisms and rationale behind our findings, it’s essential to consider the interplay between various factors influencing HL among HAs. The observed higher HL scores among those with higher education levels, for instance, can be attributed to their enhanced ability to process complex information, navigate healthcare systems, and adopt beneficial health behaviors. Similarly, the disparities between urban and rural residents may stem from differential access to healthcare facilities, social institutions, and communication networks, which collectively impact health information acquisition and skill development. Understanding these underlying mechanisms is crucial for designing targeted interventions that address specific needs and challenges within different demographic groups.

 This research is subject to certain limitations, primarily stemming from the cultural and socioeconomic diversity across East Azarbaijan province, which may introduce potential biases and affect the generalizability of the findings. While efforts were made to mitigate these limitations through appropriate sample size calculation and random sampling methods, future studies should explore these variables in greater detail to enhance the robustness of HL research in similar contexts. Additionally, the study’s reliance on self-reported data may introduce response bias, and the cross-sectional design precludes causal inferences. Future research should focus on longitudinal studies to examine the long-term impact of HL interventions on HAs and their communities. Qualitative studies could provide deeper insights into the lived experiences of HAs and the barriers they face in promoting HL. Furthermore, there is a need to explore the effectiveness of different training modalities and communication strategies in enhancing the HL of HAs, taking into account the cultural and linguistic diversity of the population.

 The findings of this study have several practical implications for policymakers and healthcare practitioners. There is an urgent need to prioritize HL initiatives targeting household HAs, particularly those with lower education levels, rural residents, and older adults. These initiatives should focus on providing tailored education and training programs that enhance their ability to access, understand, evaluate, and apply health information effectively. Additionally, policymakers should invest in strengthening healthcare infrastructure and communication networks in rural areas to reduce disparities in HL and promote equitable access to health information for all communities.

## Conclusion

 Our findings emphasize the urgent need to assess and enhance the HL of household HAs in a developing country, like Iran. Notably, a significant portion of participants exhibited varying levels of HL, with 8% classified as having poor HL. This trend underscores the vital role that HAs play in promoting HL and facilitating individual self-care within their communities. To empower these ambassadors, it is crucial to implement innovative and targeted strategies that effectively boost their HL. By equipping them with the necessary knowledge and skills, we can enhance their capacity to support and educate others, leading to improved health outcomes across society. Strengthening the HL of household ambassadors not only benefits individuals but also fosters healthier communities, ultimately contributing to the overall well-being of our population.

## Competing Interests

 The authors declare no conflict of interest.

## Data Availability Statement

 Data are available per request.

## Ethical Approval

 The research was approved by the medical ethics board in Tabriz University of Medical Sciences by the institutional review board (Ethics approval No. IR.TUMS.1402.023). The researcher assured the respondents of the confidentiality of the collected information.
